# Aggressive high-grade urothelial carcinoma transforming into enteric-type adenocarcinoma: a case report

**DOI:** 10.1016/j.radcr.2025.06.056

**Published:** 2025-08-05

**Authors:** Vaishnavi Dongare, Manjusha Mahakalkar, Shalini Moon

**Affiliations:** Department of Obstetrics and Gynecological Nursing, Smt. Radhikabai Meghe College of Nursing, Sawangi (Meghe), Wardha, Datta Meghe of Medical Sciences, Maharashtra, India

**Keywords:** Urothelial carcinoma, Enteric-type adenocarcinoma, Case report, Chemotherapy, Radiotherapy

## Abstract

Bladder cancer, a malignancy with high morbidity and mortality, predominantly affects older adults, with urothelial carcinoma as the most common histological type. However, enteric-type adenocarcinoma arising from high-grade urothelial carcinoma is an exceptionally rare and aggressive variant.

This report details the case of a 44-year-old woman diagnosed at Rural Hospital. She presented with hematuria and frequent micturition, leading to a diagnosis of high-grade urothelial carcinoma with villoglandular differentiation. Despite multiple chemotherapy cycles (Gemcitabine-Carboplatin, followed by MVAC), the tumor progressed. Imaging revealed extensive invasion, necessitating radical anterior exenteration, including cystectomy, hysterectomy, and ileal conduit construction. Histopathology confirmed transformation into enteric-type adenocarcinoma with perineural and lymphovascular invasion but no lymph node metastases. Adjuvant radiotherapy was administered to mitigate recurrence risk.

This case underscores the complexities of managing aggressive bladder carcinoma with histological transformation and highlights the need for a multimodal treatment strategy. Individualized approaches integrating surgery, chemotherapy, and radiotherapy are crucial. Further research is needed to establish standardized protocols and identify biomarkers for early detection and targeted therapy.

## Introduction

Urothelial carcinoma, the most common subtype of bladder cancer, arises from the bladder’s urothelial cells and predominantly affects older adults. It is frequently associated with smoking and chemical exposure, both of which contribute to its pathogenesis [1]. High-grade urothelial carcinoma exhibits aggressive behavior, a high risk of progression, and a propensity for histological transformation. This report presents a unique case of urothelial carcinoma transforming into enteric-type adenocarcinoma, an exceedingly rare occurrence. Such transformations pose significant diagnostic and therapeutic challenges, often requiring multimodal management strategies involving surgery, chemotherapy, and radiotherapy. This case underscores the need for personalized treatment approaches to improve patient outcomes, highlighting the importance of further research into the mechanisms driving histological transformation and therapeutic resistance [2].

## Case study

A 44-year-old female was admitted to Rural Hospital, with primary concerns of hematuria and increased urinary frequency persisting for 2 and a half years. Her family history reveals that her maternal grandmother had cervical cancer. She has no significant comorbidities in her medical history.

**Fig. 1:**
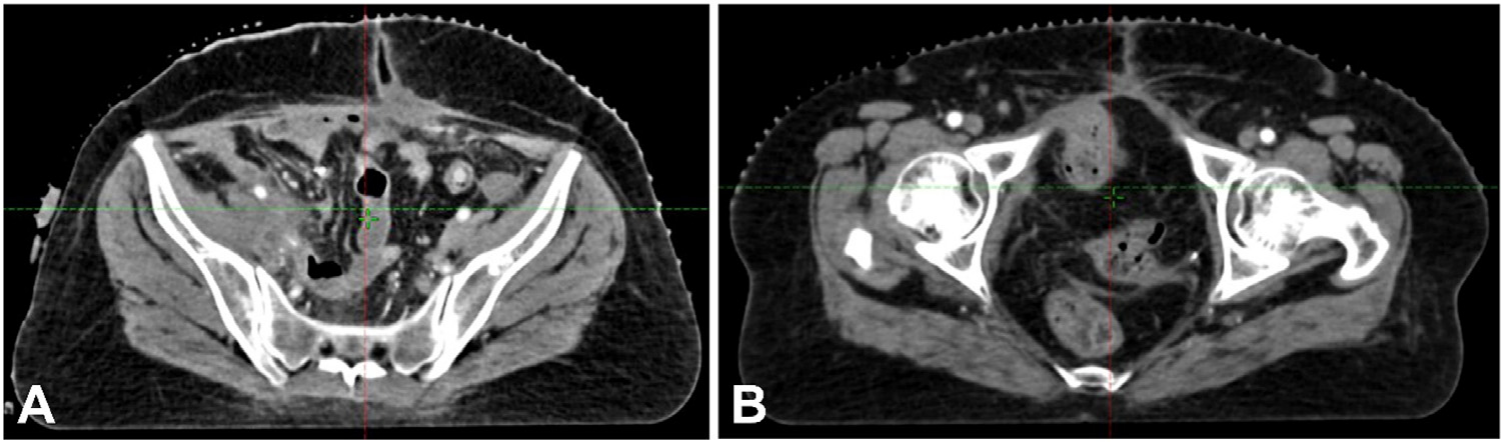
Diagnostic CT images showing pelvic mass involving the bladder.

**Fig. 2:**
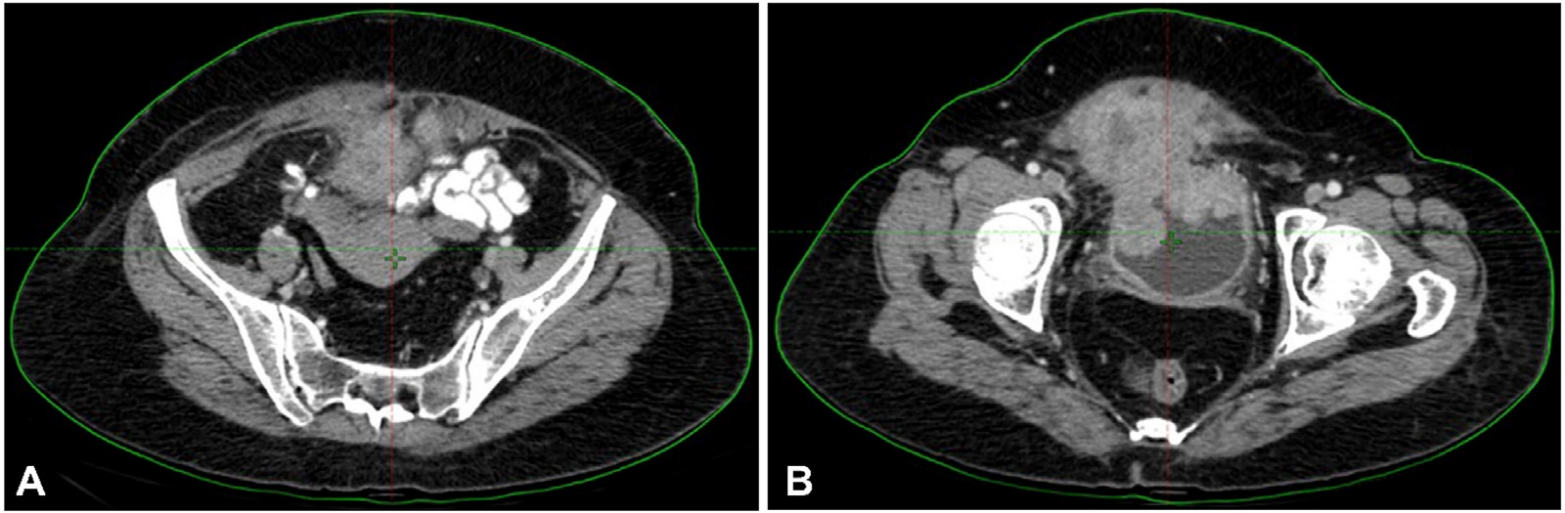
Diagnostic CT images showing pelvic mass involving the bladder.

**Fig. 3:**
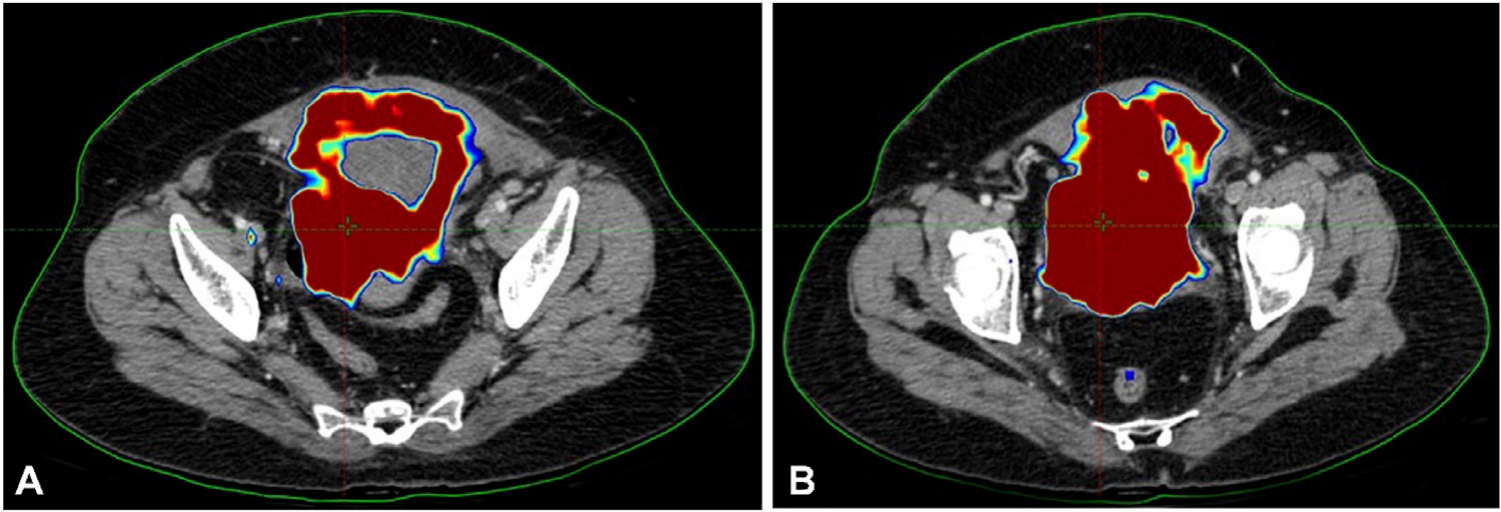
Radiation dose distribution planning on CT for bladder carcinoma.

**Fig. 4:**
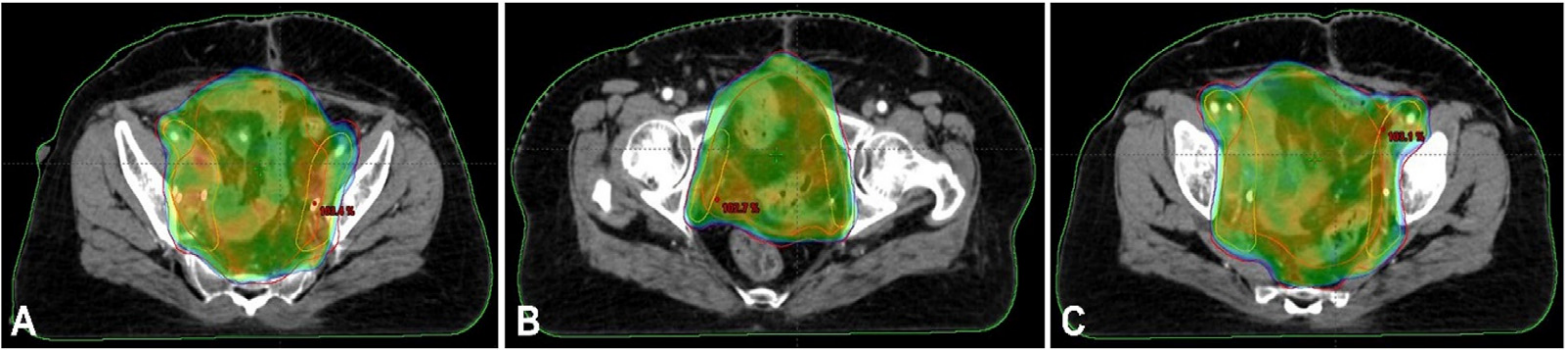
PET-CT based radiation therapy planning for bladder carcinoma.

Initial diagnostic evaluations, including imaging and biopsy, confirmed high-grade urothelial carcinoma with villoglandular differentiation. Physical examination and imaging revealed an 8.1 × 7.4 × 5.7 cm soft tissue mass with calcified foci extending irregularly toward the bladder's apex, near the uterine wall but not invading adjacent intestinal loops. A PET-CT scan indicated significant invasion, confirming the persistence of disease despite initial chemotherapy. Pathological analysis validated the diagnosis of high-grade urothelial carcinoma invading the lamina propria (Fig. 1, Fig. 2, Fig. 3, Fig. 4).

The patient underwent 4 cycles of AUC2 Gemcitabine + Carboplatin chemotherapy. However, due to tumor progression, a therapeutic reevaluation led to the administration of MVAC chemotherapy. Subsequent PET-CT scans showed further tumor growth (9.6 × 9.2 × 8.6 cm), necessitating radical anterior exenteration, including ileal conduit construction, radical cystectomy, and complete abdominal hysterectomy with bilateral salpingo-oophorectomy.

Histopathological analysis of the resected mass confirmed transformation into enteric-type adenocarcinoma with perineural and lymphovascular invasion but no lymph node metastases. Consequently, adjuvant radiation therapy was administered to enhance local disease control.

### Timeline

The patient initially presented with hematuria and increased micturition. Following diagnostic evaluation, she was diagnosed with high-grade urothelial carcinoma. She underwent 4 cycles of AUC2 Gemcitabine and Carboplatin chemotherapy. However, PET-CT results indicated persistent tumor activity and invasion, leading to the decision for surgical intervention. A radical cystectomy with anterior exenteration was performed, and postsurgical histopathological analysis revealed enteric-type adenocarcinoma. Subsequently, adjuvant radiotherapy was initiated.

### Diagnostic assessment

The patient's diagnosis was established through clinical examination, pathology, and PET-CT imaging. A significant challenge encountered during treatment was resistance to the initial chemotherapy regimen. The final diagnosis revealed high-grade urothelial carcinoma with enteric-type adenocarcinoma features.

### Therapeutic interventions

The patient initially received 4 cycles of Gemcitabine and Carboplatin as first-line chemotherapy. However, due to disease progression, she was transitioned to the MVAC regimen as second-line therapy. Surgical intervention included a radical cystectomy, ileal conduit construction, and abdominal hysterectomy. Postsurgery, adjuvant radiotherapy was administered to the surgical bed and high-risk lymphatic regions.

### Follow-up and outcomes

At follow-up visits, no recurrence was detected, indicating a positive treatment outcome. The patient was monitored through periodic imaging and clinical assessments. She tolerated radiation therapy well, with no significant complications reported. At the last follow-up, the patient remained disease-free.

## Discussion

This case presents an exceedingly rare transformation of high-grade urothelial carcinoma into enteric-type adenocarcinoma, a phenomenon that remains poorly understood. While histological transformation in bladder cancer has been reported, the shift to an enteric-type variant is uncommon and poses significant diagnostic and therapeutic challenges [3]. Unlike conventional high-grade urothelial carcinoma, which often responds to platinum-based chemotherapy, this case exhibited resistance to both Gemcitabine-Carboplatin and MVAC regimens, suggesting underlying molecular alterations that may drive chemotherapy resistance [4]. The role of targeted therapies in such cases remains an area of active research.

Bladder cancer predominantly affects older adults, yet this patient was diagnosed at 44 years of age, raising questions about genetic or environmental risk factors. Although no direct hereditary link was established, a family history of cervical cancer suggests a possible predisposition to malignancy. Studies have indicated that early-onset bladder cancer may be associated with distinct genetic mutations, warranting further investigation through next-generation sequencing and molecular profiling to identify genetic contributors to histological transformation [5].

Despite chemotherapy, tumor progression and invasion persisted, necessitating radical anterior exenteration. Histopathological findings confirmed perineural and lymphovascular invasion, yet the absence of lymph node metastasis highlights the unpredictable nature of transformed urothelial carcinoma. The role of adjuvant radiotherapy in such cases remains underexplored, though emerging evidence suggests its potential to improve local disease control [6]. In this case, it was integrated into treatment to reduce recurrence risk, supporting the growing evidence favoring multimodal management in rare bladder cancer subtypes [7].

This case underscores the necessity of individualized treatment based on tumor histology and molecular characteristics. Given the failure of conventional chemotherapy, exploring precision oncology approaches such as biomarker-driven therapies and immune checkpoint inhibitors is crucial [8]. Targeted molecular inhibitors may offer a more effective strategy for bladder cancer cases with enteric differentiation.

Molecular profiling and biomarker identification are crucial in understanding the genetic drivers behind the transformation of urothelial carcinoma, which can facilitate the development of targeted therapies and lead to improved treatment outcomes [9]. Due to the rarity of enteric-type adenocarcinoma in the bladder, there is a pressing need for multicenter studies and case series to establish standardized, evidence-based treatment guidelines. Furthermore, considering the limited effectiveness of conventional chemotherapy, the role of immunotherapy—particularly immune checkpoint inhibitors—and novel targeted molecular therapies should be thoroughly explored as promising alternatives for better disease management [10].

## Conclusion

A multimodal therapeutic approach is essential for managing aggressive high-grade urothelial carcinoma with intestinal differentiation, as demonstrated by this instance. Due to the complex nature of this malignancy, which is marked by rapid growth and resistance to conventional treatments, therapy must be individualized and comprehensive. Since enteric-type adenocarcinoma from high-grade urothelial carcinoma is uncommon, further research is required to comprehend its pathogenesis, find biomarkers for early identification, and develop evidence-based treatment recommendations. In order to provide a comprehensive therapeutic approach, this case also encourages collaboration among oncologists, radiologists, surgeons, and pathologists. Lastly, enhancing treatment options and understanding these rare and aggressive cancer subtypes may significantly increase patient survival and quality of life.

## Patient perspective

The patient experienced significant physical and emotional challenges throughout her treatment journey. Initially, she struggled with anxiety and uncertainty regarding her diagnosis, particularly given the aggressive nature of the disease and its resistance to chemotherapy. The need for radical anterior exenteration and an ileal conduit profoundly impacted her daily life, requiring major adjustments in self-care and mobility.

Emotionally, she expressed distress over the changes in her body image and concerns about social stigma associated with urinary diversion. However, she found support through counseling and peer support groups, which helped her navigate these challenges.

Regarding treatment decision-making, the patient played an active role, thoroughly discussing available options with her medical team. Although initially hesitant about extensive surgery, she ultimately opted for a multimodal approach after understanding the risks of disease progression. She expressed gratitude for the multidisciplinary care received and emphasized the importance of clear communication between patients and healthcare providers in making informed treatment choices.

## Informed consent

Informed consent was obtained from the patient for publication of this case report.

## Significance of the study

This study provides insights into the rare transformation of high-grade urothelial carcinoma into enteric-type adenocarcinoma, emphasizing the need for a multidisciplinary approach in managing aggressive bladder cancers. The findings highlight the importance of individualized treatment strategies and early detection in improving patient outcomes.

## Limitations

This case report is limited by its single-patient focus, restricting the generalizability of findings. Additionally, the absence of molecular or genetic profiling limits a deeper understanding of the mechanisms driving histological transformation. Future studies should investigate the genetic and molecular characteristics of such rare bladder cancer subtypes to develop targeted, personalized therapies. Long-term follow-up data would also strengthen the understanding of treatment outcomes and recurrence patterns.

## Patient consent

Written informed consent was obtained from the patient for the publication of this case report, including relevant clinical details and images. The patient understands that their personal information will be anonymized to ensure confidentiality.
